# Managing emotions in the age of political polarization: A randomized controlled trial comparing mindfulness to cognitive reappraisal

**DOI:** 10.21203/rs.3.rs-3947259/v1

**Published:** 2024-03-28

**Authors:** Hadley Rahrig, Polina Beloboradova, Christina Castro, Kayla Sabet, Melina Johnson, Orion Pearce, Kirk Warren Brown

**Affiliations:** 1 Department of Psychology, University of Wisconsin-Madison, Madison, WI, 53703, United States of America; 2 Department of Psychology, Virginia Commonwealth University, Richmond, VA, 23284, United States of America; 3 Health and Human Performance Lab, Carnegie Mellon University, Pittsburgh, PA, 15213, United States of America

## Abstract

Emotional appraisals of political stimuli (e.g., videos) have been shown to drive shared neural encoding, which correspond to shared, yet divisive, interpretations of such stimuli. However, mindfulness practice may entrain a form of emotion regulation that de-automatizes social biases, possibly through alteration of such neural mechanisms. The present study combined a naturalistic neuroimaging paradigm and a randomized controlled trial to examine the effects of short-term mindfulness training (MT) (n = 35) vs structurally equivalent Cognitive Reappraisal training (CT) (n = 37) on politically-situated emotions while evaluating the mechanistic role of prefrontal cortical neural synchrony. Participants underwent functional near-infrared spectroscopy (fNIRS) recording while viewing inflammatory partisan news clips and continuously rating their momentary discrete emotions. MT participants were more likely to respond with extreme levels of anger (*odds ratio* = 0.12, *p* < .001) and disgust (*odds ratio* = 0.08, *p* < .001) relative to CT participants. Neural synchrony-based analyses suggested that participants with extreme emotion reactions exhibited greater prefrontal cortical neural synchrony, but that this pattern was less prominent in participants receiving MT relative to CT (CT > MT; channel 1 ISC = .040, *p* = .030).

American citizens have shown an upward trend in political polarization, characterized in part by the perceived division of moral values along party lines ^1^. This moralization of political identity has contributed to the escalation of negative emotions (e.g., fear, anger, and hatred) directed towards political outgroup members, which further reinforce partisan identities ^2^ and strengthen intergroup partisan prejudices. ^3^ Although negative emotions are potent motivators of political intolerance, these emotions are nevertheless subject to regulation ^4,5^. Accordingly, there has been a recent increase in research examining the regulation of political intergroup emotions ^6^; however, interventions designed to promote emotion regulation have yielded mixed results ^7–9^. The present study explored the effects of mindfulness—an emotion regulation skill rarely studied in the context of political polarization—on partisan intergroup emotions. Probing neural mechanisms associated with partisan information processing, we further examined if mindfulness altered prefrontal cortical neural synchrony while viewing inflammatory political videos.

## How socio-political systems influence emotion

While Western psychology has historically conceptualized emotion as an individual experience, emerging theories with a basis in systems ecology suggest that emotion operates as a systems-level phenomenon, such that the meaning of emotion is dependent on social, cultural, and institutional contexts ^10–12^. An implication of these theories is that the human brain interprets cultural signals (e.g., language and gestures) as a means of establishing shared understanding among multiple individuals. This mechanism enables emotions to be felt at the group level, thereby motivating important cooperative behaviors ^13^. However, such “group-based emotions” can also serve as the basis for intergroup prejudices when emotions become negatively directed towards other social groups ^1,14^.

How do humans navigate an environment of complex socio-political systems and high-stakes intergroup relations? Neuroimaging research suggests that humans have the capacity to establish a ‘generalized shared reality’ ^15^ through the alignment of internal, neural processes ^15–17^. This brain-to-brain coupling—otherwise referred to as interpersonal neural synchrony—occurs when the neural activation of two or more individuals becomes temporally correlated, leading to the synchronization of ongoing perceptions and cognitions ^18^. Notably, interpersonal neural synchrony is not limited to dyads, but rather appears to scale to the level of social groups ^19–22^. In general, participants who are shown complex naturalistic stimuli (e.g., audiovisual films) will exhibit robust within-group synchrony ^23^; however, when stimuli contain divisive political content, the meaning of the stimuli—and by extension, the trajectory of neural responses—becomes dependent on a participant’s political affiliations and ideologies ^20,21^.

By way of illustration, Leong et al. (2020) observed that neural synchrony within the dorsomedial prefrontal cortex (dmPFC)—a region implicated in narrative interpretation—significantly differed between liberal and conservative participants who viewed the same political videos. This phenomenon, which Leong et al. (2020) refer to as “neural polarization”, likewise predicted attitude polarization, such that participants who were more neurally “in sync” with their political ingroup were more likely to shift their attitudes to match their own party’s position. While this evidence underscores that intergroup prejudices are powerfully influenced by emotional processes, it also suggests that partisan biases may be altered through the regulation of such emotions.

## Interventions for Intergroup Emotion Regulation

Evidence is clear that partisan information can provoke strong emotional reactions associated with threats to personal identity and moral ideology ^2^; however, this picture is incomplete without considering *emotion regulation* in the scope of partisan politics ^24^. Among emotion regulation techniques, cognitive reappraisal has been framed as a generally adaptive strategy in terms of affective, physiological, and social outcomes ^25^. By definition, cognitive reappraisal involves the deliberate modulation of thoughts ^26^, and in a political context, may manifest as rationalization of the status quo ^27^, minimization of perceived impact, or reframing of events as meaning-making opportunities ^28^. While research suggests that cognitive reappraisal is a common and effective practice for the management of chronic political stress ^8^, cognitive reappraisal is not without its limitations. Reappraisal requires deliberate manipulation of thoughts, which – in terms of cognitive operations – is relatively time-consuming and difficult to deploy in the midst of a distressing event ^29^. Consequently, the ability to engage in reappraisal may fail in high-arousal contexts ^30^, and even when effective, may fail to address identity-based motivations that sustain intergroup conflict ^6^. These limitations have invited the investigation of alternative mental practices to ameliorate intergroup conflict.

*Mindfulness*, a concept with roots in Buddhism, has been defined as a mental state or mental quality of attention to present-moment emotions, thoughts, and sensations with an orientation of non-judgemental acceptance ^31^. According to an innatist perspective ^32^, individuals vary in their natural capacity for mindfulness ^31^. Yet mindfulness skills can be enhanced through standardized secular training programs (spanning days or weeks in length), which guide participants through formal mindfulness practices ^33^. To date, very few studies have tested the effects of mindfulness-based interventions on political intergroup emotions ^34–37^. One such study, situated in the Israeli-Palestinian conflict, observed that mindfulness training increased support for conciliatory policies, and this effect was uniquely attributed to the effect of mindfulness on reducing negative emotions and perceptions of threat ^34^. Similar findings were reported in the context of a highly polarized U.K. electorate, with results showing that negative attitudes about partisan rivals were reduced following mindfulness training ^35^. While initial findings are promising, this line of research is still nascent, and it remains unclear if and how mindfulness targets emotion-related processes implicated in intergroup prejudice.

## Mindfulness and Intergroup Emotions

Over three decades of research has documented the impact of mindfulness ^38^, with research predominantly focusing on the application of secular mindfulness practices for promoting wellbeing at the individual level. However, contemplative theories have long acknowledged the value of mindfulness for interpersonal purposes ^39^, a position which has been corroborated by emerging empirical research ^40–42^. Initial studies suggest that trait mindfulness and mindfulness-based practices may promote compassionate behavior, reduce aggressive retaliation ^43^, and may even attenuate intergroup bias ^44–46^. While at first glance it may seem unclear why the intrapersonal benefits of mindfulness would extend to intergroup relations, these effects may be explained by interrogating well-known mechanisms of mindfulness-based practices.

Unlike other forms of emotion regulation that aim to alter the expression or duration of emotion, mindfulness indirectly supports emotional functioning by fostering *meta-awareness*, defined as the ability to recognize the experience of having an emotional reaction (among other mental events) as it occurs in real time ^47^. Meta-cognitive models of mindfulness posit that meta-awareness supports emotion regulation through a number of possible pathways ^48–50^. For example, greater awareness of emotional experiences may operate as a form of exposure, thereby reducing the intensity of emotions through inhibitory learning ^51^. Moreover, recognizing emotions as they initially arise can disrupt habitual cognitive elaborations (i.e., rumination, cognitive distortions) that tend to perpetuate and amplify emotional distress ^52^, and absent such habitual responses, may enable more flexible selection of emotion regulation strategies ^53,54^. A notable consequence of such meta-awareness is that emotional states may be observed with a sense of psychological distance—otherwise referred to as disidentification—such that emotions no longer feel objectively true or experientially fused with one’s sense of self ^55^. In the context of partisan triggers, meta-awareness and disidentification may function synergistically to disrupt habitual, biased responses (i.e., biased perceptions of partisan others) and mitigate perceived threat to personal ideologies.

This meta-cognitive framework receives support from converging neuroimaging research, although very few neuroimaging studies have tested these theories in the social domain (e.g., Kirk et al., 2016; Laneri et al., 2017; Quaglia et al., 2019). Meta-awareness, which theoretically extends from improvements in executive functioning, has been linked to enhanced activity in regions of the frontoparietal control network (FPCN) (e.g., dorsal and ventral portions of the lateral prefrontal cortex) following mindfulness training ^48,59^. Such improvements in executive functioning are thought to stabilize present-moment attention, and contribute to the suppression of the default mode network (DMN) regions (e.g., dorsomedial and ventromedial PFC, posterior cingulate), broadly associated with functions such as self-reflection, narrative processing, and interpretation of social content ^60,61^. In the context of mindfulness, such DMN down-regulation has been associated with disidentification from experience; however, this mechanism may also have unexplored implications for interpersonal neural synchrony. Evidence shows that interpersonal neural synchrony is commonly localized to the core regions of the DMN, namely dorsal and ventromedial PFC regions, and is especially prominent when naturalistic stimuli evoke negative emotions ^23,62^. Emerging theories suggest that the DMN may play a crucial role in synchronizing mental states, given its dual involvement in the integration of self knowledge and the interpretation of ongoing social cues ^17^. Whether or not mindfulness training can alter this neural mechanism has yet to be experimentally tested.

## The Present Study: Rationale for an Interdisciplinary Approach

The current project adopts a research approach described by Wilson-Mendenhall and Holmes (2023) as *use-inspired basic research,* which aspires to the complementary integration of basic and applied research to simultaneously deepen affective science while advancing positive social change ^63^. In alignment with this framework, we pursued a research paradigm poised to clarify the mechanisms of partisan intergroup emotions while investigating the applied use of validated interventions to address such emotions. This overarching goal was accomplished through the synthesis of a neural synchrony approach, achieved using functional near-infrared spectroscopy (fNIRS), and a randomized controlled trial comparing brief mindfulness training against cognitive reappraisal training. Specifically, we posed two mutually informative research questions. First, we sought to examine how discrete emotions modulated neural representations of politically partisan video content. Second, we aimed to determine if negative emotions, which have been linked to partisan biases, could be reduced by training in mindfulness versus cognitive reappraisal. Connecting the first to the second aim, this intervention also served as an experimental probe to examine whether neural synchrony was differentially expressed by participants who completed either mindfulness training or cognitive reappraisal training. Finally, as an ancillary aim, we examined whether mindfulness training compared to cognitive reappraisal training influenced biased attitudes about political outgroup members.

Participants, who were liberal-leaning and Democratic voting community adults, were randomized to receive one of two validated 14-day stress reduction programs: Mindfulness (*n* = 35) or a structurally equivalent Active Coping program (*n* = 37) emphasizing cognitive reappraisal ^64^. Prior to randomization and immediately following training, participants completed a laboratory session during which neuroimaging data were collected via functional near-infrared spectroscopy (fNIRS), a non-invasive neuroimaging device designed to record brain activity as blood-oxygen-level dependent (BOLD) signal. FNIRS acquisition took place while participants completed a novel, naturalistic viewing paradigm, during which participants viewed a series of politically partisan videos and rated their momentary emotional reactivity across five emotions: joy, anger, fear, disgust, and sadness. From this data, we were able to infer neural representations via intersubject correlation and intersubject representational similarity analysis, and evaluate the interrelations between such neural signals and emotions, as well neural and subjective outcomes of mindfulness training.

## Results

### Neural Responses to Partisan Content Reflect Discrete Emotion States

**Figure 1.**
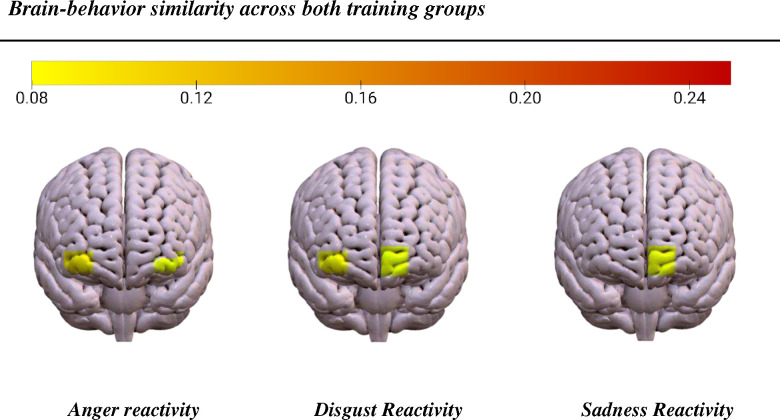
IS-RSA results collapsed across both groups at post-training using the AnnaK similarity model. Anger scores corresponded to bilateral ventrolateral PFC synchrony (channel 4, *r* = .09, *p* = .041; channel 19, *r* = .12, *p* = .013). Disgust scores corresponded to synchrony within left ventromedial PFC (channel 11, *r* = .082, *p* = .047) and the right ventrolateral PFC (channel 19, *r* = .10, *p* = .039). Sadness scores corresponded to synchrony within the left ventromedial PFC (channel 11, *r* = .09, *p* = .02).

A series of IS-RSAs were computed to identify regional sources of neural signal that contributed to shared emotion response to political videos, measured here as anger, disgust, sadness, fear and joy reactivity. First, subject-by-subject intersubject similarity matrices were constructed for self-reported anger reactivity and fNIRS time courses across each of 20 fNIRS channels, distributed across regions of the prefrontal cortex (PFC). A paired-sample t-test and scatterplot examination indicated that an Anna Karenina (AnnaK) similarity model was a superior fit for structuring the data, such that participants with relatively high anger scores exhibited greater time course neural similarity while those with relatively low anger scores exhibited greater temporal idiosyncrasy. The correlation coefficients between the upper triangles of the brain and behavior similarity matrices were tested non-parametrically (5000 permutations; *p* < .05). This procedure was repeated for all discrete emotion scores. Using the AnnaK similarity model, IS-RSA identified a significant relationship between anger reactivity and intersubject neural synchrony in the bilateral ventrolateral PFC (channel 4, *r* = .09, *p* = .041; channel 19, *r* = .12, *p* = .013). A significant relationship between disgust reactivity and intersubject neural synchrony was identified within the left ventromedial PFC (channel 11, *r* = .082, *p* = .047) and the right ventrolateral PFC (channel 19, *r* = .10, *p* = .039). Sadness also modulated intersubject neural synchrony within the left ventromedial PFC (channel 11, *r* = .09, *p* = .02). Significant brain-behavior relations for anger, disgust, and sadness are displayed in Figure 1. The AnnaK similarity model also revealed a negative relation between joy similarity scores and neural time signatures within the right vmPFC (channel 15, *r* = −.099, *p* = .017), suggesting that participants who reacted with relatively greater joy showed greater temporal idiosyncrasy. Permutation tests did not reveal any relationships between fear and neural similarity within any regions.

### Impact of Mindfulness vs. Cognitive Reappraisal Training on Emotion Reactivity

**Figure 2.**
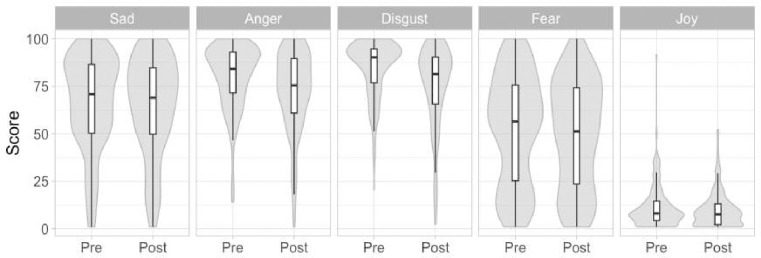
Boxplots of emotion reactivity measures in the pre- and post-training periods

Emotional reactions to the stimuli were skewed toward greater intensity for all emotions except fear, with numerous participants showing such extreme reactions as zero joy and the highest possible level of sadness, anger, and disgust (see Fig. 2).

The results of Zero-inflated Gaussian mixed models indicated that mindfulness training led to overall greater joy (*b* = 0.23, *p* = .028), however it also increased the odds of selecting zero joy (*odds ratio* = 0.16, *p* < .001). Similarly, MT participants were significantly more likely to select the highest values of anger (*odds ratio* = 0.12, *p* < .001) and disgust (*odds ratio* = 0.08, *p* < .001). MT did not affect less extreme anger and disgust responses (*p*’s > .065). There was no effect on either extreme (*p* = .238) or less extreme sadness responses (*p* = .606) and no effect on fear (p = .063). Detailed model results are presented in Tables 1 and 2.

### Comparing Neural Synchrony Between Mindfulness and Cognitive Reappraisal

#### Between-group Intersubject Correlation Analysis

**Figure 3.**
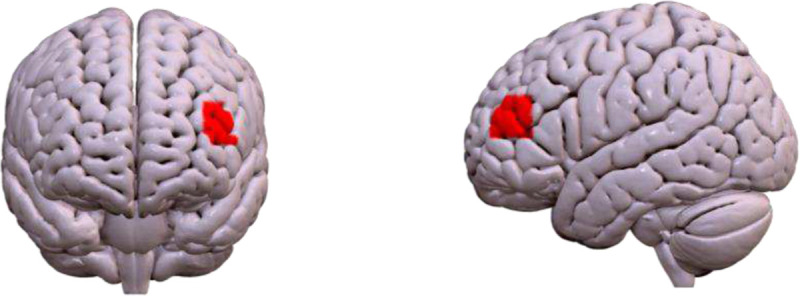
Anterior (left) and sagittal (right) view of between-group intersubject correlation effect (CT > MT) localized to the left dorsolateral prefrontal cortex (channel 1; ISC = .040, *p* = .030).

An intersubject correlation (ISC) analysis was conducted to determine if emotion-modulated neural synchrony (identified by IS-RSA) varied between participants who received mindfulness and those who received cognitive reappraisal training. Thus, ISC was computed using fNIRS signals recorded during the post-training naturalistic viewing paradigm. Initial similarity analyses detected within-group neural synchrony within 25–95% of analyzed channels. A leave-one-out approach was used to estimate individual-level ISC values for each channel. Between-group comparisons were tested using subject-wise permutations over 5000 iterations.^65^ This approach identified channels with significant within- versus between-group synchrony while controlling for false positive rate (FPR). Two-sample ISC analysis indicated between-group differences in neural synchrony localized to the left dorsomedial PFC (channel 1; ISC = .040, *p* = .030). Relative to mindfulness participants, control participants exhibited greater neural synchrony within the dorsolateral PFC, a region associated with socio-emotional interpretation, when viewing emotionally provocative political videos (see Fig. 3).

#### Within-group analysis of brain-behavior relationships

**Figure 4.**
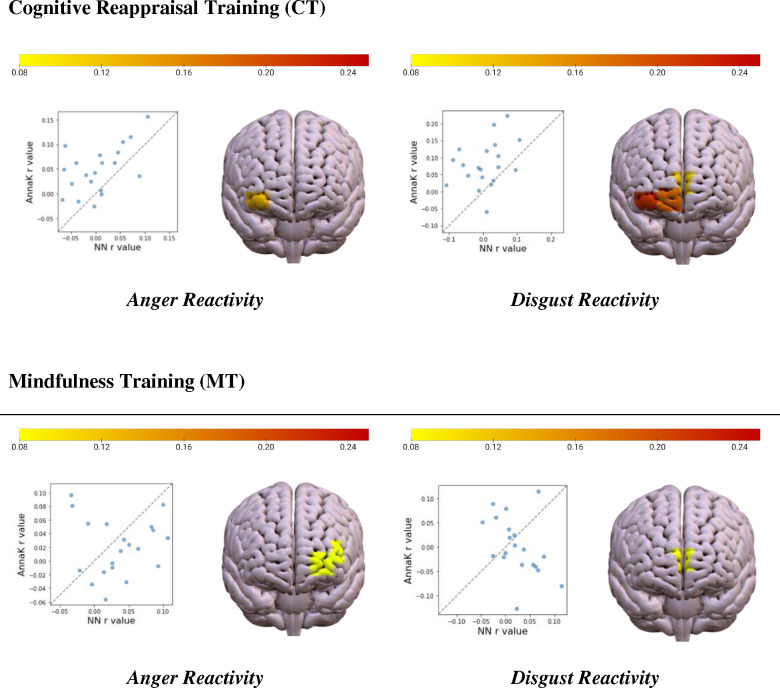
IS-RSA within-group results specific to anger and disgust reactivity. Scatterplots show R values for each channel calculated using an AnnaK and NN similarity structure, indicating that AnnaK similarity structure was a superior fit to the CT data and NN similarity structure was a superior fit to the MT data. CT participants showed a significant relationship between anger reactivity and right vlPFC synchrony (Channel 19, *r* = .157, *p* = .047), and disgust and neural similarity within the right vlPFC and vmPFC (Channel 12, *r* = .152, *p* = .033; Channel 13, *r* = .197, *p* = .021; Channel 19, *r* = .223, *p* = .0034). MT participants showed a relationship between anger and neural similarity within the left ventromedial PFC (Channel 5, *r* = .10, *p* = .037; Channel 6, *r* = .11, *p* = .031), and disgust and neural similarity within the vmPFC (Channel 12, *r* = .115, *p* = .021).

To examine whether mindfulness training and cognitive reappraisal training differentially influenced the mechanisms by which emotions modulated neural encoding of politically partisan material, intersubject representational similarity analysis (IS-RSA) was repeated using the above protocol with the modification that participants receiving mindfulness training (MT) and cognitive reappraisal training (CT) were analyzed separately. Notably, dividing analysis by group increased the magnitude of brain-behavior relationships across all emotion categories. Moreover, MT and CT exhibited different patterns of neural similarity in terms of brain-behavioral similarity structure. For example, CT participants showed a significant relationship between anger reactivity and right ventrolateral PFC synchrony (Channel 19, *r* = .157, *p* = .047), with high anger participants showing greatest neural similarity and low anger participants showing greatest idiosyncrasy (i.e., an Anna Karenina similarity structure). In contrast, MT participants showed a relationship between anger and neural similarity within the left ventromedial PFC (Channel 5, *r* = .10, *p* = .037; Channel 6, *r* = .11, *p* = .031) such that low anger participants were neurally synchronized with other low anger participants and high anger participants were neurally synchronized with other high anger participants (i.e., a Nearest Neighbor Similarity structure). This pattern of group-based neural similarity was found across all measured discrete emotions, with the exception of fear, which was not significantly related to neural synchrony for either group. IS-RSA results specific to anger and disgust reactivity are illustrated in Figure 4, and the complete report of IS-RSA findings by training group are displayed in Table 3.

### Impact of Training on Explicit Intergroup Attitudes

We used the Mann Whitney U test to disclose whether MT and CT participants exhibited different levels of affective prejudice and social distancing to both ingroup (democrats) and outgroup members (republicans). The non-parametric test was selected because the data were not normally distributed. Instead of observed values, the Mann Whitney U test uses observation ranks to evaluate whether two samples come from the same or different populations. We did not find statistically significant differences between CT and MT groups in any of intergroup attitudes (*p*’s > .166).

## Discussion

The present study offers a novel approach to testing the effectiveness of mindfulness as a theoretically and empirically supported means for reducing political intergroup biases while probing the mechanisms through which such biases may be attenuated. Results reported here suggest that when viewing inflammatory partisan media, mindfulness training may amplify negative emotions such as anger and disgust while supporting coping through increased positive appraisal. Divergent emotional responses between groups may reflect training-based changes in the neural encoding of videos, as suggested by training-based differences in neural synchrony. Herein, we summarize our findings emerging from this approach and elaborate upon its strengths and weaknesses. We likewise reflect upon opportunities to advance this research through interdisciplinary methodologies, and critically examine mindfulness training as a tool to buffer the impact of political polarization.

### Intersubject Neural Synchrony is Modified by Discrete Emotions

We conducted a series of intersubject representational similarity analyses (IS-RSAs) to evaluate how discrete emotions, particularly negative emotions, modulate neural synchrony among participants. In other words, we sought to understand how emotions contributed to shared neural encoding of politically partisan news clips, a phenomenon that has previously been associated with polarized attitudes and moral-emotional appraisals ^19–21,66^. Analysis of post-training fNIRS recordings suggested that negative emotions were modulated by prefrontal cortical neural synchrony. Anger reactivity was associated with neural synchrony within the ventrolateral PFC, disgust was associated with ventrolateral PFC and ventromedial PFC synchrony, and sadness was associated with ventromedial PFC synchrony. These findings broadly align with prior research linking the ventromedial PFC (vmPFC) and ventrolateral PFC (vlPFC) to emotion representation and regulation ^67–70^.

The vmPFC is centrally involved in the generation of affective meaning, and has been mechanistically linked to the construction of interpersonal anger and disgust ^71,72^, as well as perpetuation of socio-cognitive biases (Lyu et al., 2023). Similarly, our research suggests that the vmPFC is involved in encoding emotional responses to politically partisan videos, which may in turn shape how partisans collectively interpret such information. Like the vmPFC, the vlPFC is associated with emotion generation and regulation ^70^ and research demonstrates that the right and left vlPFC may be differentially linked to action- and avoidance-based anger coping strategies ^73,74^. For example, right vlPFC stimulation has been shown to attenuate negative emotions and aggression provoked by frustration and social exclusion ^74^, but in situations when individuals feel unable to express anger, right vlPFC activity may actually enhance anger rumination as an avoidance-based coping method ^73^. Such motivational nuances have important implications for anger regulation in socio-political contexts and additional research is needed to clarify the biological basis of ecologically specific emotion processes.

Performance of AnnaK behavioral similarity models suggested that participants who responded with relatively high levels of anger, disgust, and sadness showed greater neural similarity but that those with relatively lower levels of these emotions were neurally *idiosyncratic*, exhibiting neural signatures dissimilar from either high-emotion or low-emotion participants ^75^. The opposite pattern was observed for joy, such that participants scoring higher in joy showed greater neural idiosyncrasy. These findings align with previous research on the emotional basis of neural synchrony, which have shown negative, high arousal stimuli to reliably synchronize the time courses of neural activity in higher-level evaluative regions (e.g., lateral and medial PFC areas) ^62^. This phenomenon has been attributed to a fight-or-flight response to threat in which negative emotions effectively limit cognitive and behavioral repertoires ^76,77^. Based on prior neuroimaging evidence ^62^, it is plausible that negative emotions also confine neural responses in ways that are prototypical of one’s social group, particularly when it feels like the moral ideologies of one’s group are under attack ^21^.

### Effects of Mindfulness Training versus Cognitive Reappraisal

#### Discrete Emotion Reactivity

As anticipated, participants across both groups and time points reacted to politically partisan videos with strong, negatively valenced emotions. Although mindfulness training is predominantly associated with the downregulation of negative emotion ^78^, our findings suggest that short-term mindfulness training may increase the likelihood of responding to videos with extreme levels of anger and disgust. Mindfulness training is intended to heighten awareness of thoughts and feelings—negative and positive—without feeling the need to control them.^33^ Accordingly, mindfulness training may actually enhance negative emotions in complex, real-life scenarios,^79^ possibly through developing awareness of emotions that are habitually suppressed. Other research proposes that mindfulness may heighten negative emotions in moral contexts,^79,80^ and may specifically increase moral outrage when experiencing vicarious injustice.^80^ Due to the moralized nature of partisan politics, it is possible that mindfulness training similarly enhanced moral-emotional reactivity of participants in the present study. Future research may explore if negative emotions, when amplified through mindfulness, motivate prosocial behaviors such as civic engagement or antisocial behaviors such as intergroup aggression.

Mindfulness training also increased positive emotion (i.e., joy reactivity) towards experimental videos, despite the evidence indicating that these videos were unambiguously negative. Previous research offers possible explanations for why mindfulness training may enable positive appraisals of stressful events ^54^. Converging self-report and behavioral evidence suggests that mindfulness training may enhance cognitive flexibility needed to selectively disengage from negative appraisals ^53,82,83^. In turn, such de-automatization affords greater cognitive bandwidth to observe stressful events with heightened clarity. In the case of triggering political events, this may entail an individual realizing that their distress signifies a commitment to their ideological values, and that these values offer a sense of personal meaning.

#### Training-Related Differences in Neural Synchrony

ISC analysis revealed significant group differences in neural synchrony when viewing partisan videos such that within-group ISC exceeded between-group ISC within the left dorsolateral prefrontal cortex (dlPFC). In this case, divergent dlPFC synchrony was attributed to greater synchrony within the CT group relative to the MT group. Findings from our exploratory analysis, which indicated a positive association between dlPFC synchrony and positive attitudes towards ingroup partisans, suggested that the dlPFC may be implicated in reinforcing political ingroup affiliation through shared neural encoding. This relationship was found to be greater in participants trained in cognitive reappraisal (CT) relative to those trained in mindfulness (MT). In the following section we attempt to build theory around the role of the dlPFC in intergroup processes while cautiously speculating on the mechanistic role of the dlPFC in mindfulness-based interventions for political polarization.

Situated within the frontoparietal control network ^84^, the dlPFC is well-known for its role in facilitating goal-oriented cognitions and voluntary emotion regulation. While the dlPFC is considered ‘domain-general’, it has been associated with socially-specific functions (Lu & Hao, 2019; Zhou et al., 2022) such as moral decision-making ^87^ and shared interpretations of socio-emotional stimuli ^88^, and multiple studies have observed dlPFC synchronization in intergroup contexts ^89,90^. Notably, dlPFC synchrony has been implicated in both supporting *and* overriding intergroup biases. For example, the role of dlPFC synchrony in intergroup hostility has previously been documented in lab-based simulations of intergroup conflict and competition ^89,90^, with studies suggesting that alignment of dlPFC activity may facilitate leader-follower behavioral coordination ^90^, and may underlie support for hostile intergroup action ^89^. However, when motivated to overcome biases, dlPFC engagement may instead override implicit prejudices in the service of egalitarian goals ^91^. In sum, the consequences of dlPFC synchrony within any given individual or context cannot be determined without accounting for social goals and motivations.

When examining how high arousal negative emotions, specifically, anger and disgust modulated neural synchrony, the CT group exhibited right lateralized vmPFC and vlPFC neural synchrony that was modulated only by high levels of emotion, while low levels of emotions did not significantly drive shared neural activity (i.e., fitting and Anna Karenina structure). Conversely, MT participants deviated from this pattern. Anger and disgust-related neural synchrony were localized to the left and midline vmPFC, and such synchrony was equally distributed across the spectrum of emotion intensity (i.e., fitting a Nearest Neighbor structure). These group-related differences offer insight on the possible mechanisms underlying MT’s effect on extreme emotional responses motivated by political identity. While negative high arousal emotions typically enforce shared neural encoding, we speculatively suggest that bringing mindful awareness to such situations may modulate the impact of emotion on neural encoding regardless of the degree of emotion intensity.

### Limitations and Future Directions

The present study used an ecologically-valid viewing paradigm used to model the kinds of emotionally provocative content featured on social media platforms ^92^. As anticipated, participants—across both laboratory sessions and both training groups—overwhelmingly reported negative emotions towards these videos. While this paradigm approximates how partisan information is exchanged online, it is phenomenologically distinct from the face-to-face social interactions that would occur ‘in the wild’. It is reasonable that such discrepant social situations would engage divergent neuropsychological pathways.

Recognizing the complexities inherent to political intergroup emotions, we advocate for further advancement of naturalistic approaches that balance realism with experimental control. One promising approach includes the use of video-chat platforms (i.e., Zoom) for hosting face-to-face cross-ideological conversations between opposing partisans ^93^. Future research may continue to elucidate the nature of politically situated emotions and their implications for intergroup behavior by integrating video-chat with methodologies such as those reported from the current study. Indeed, functional near-infrared spectroscopy (fNIRS) may be suitably adapted for face-to-face conversations and other naturalistic settings given its portability and high motion tolerance ^94^.

As previously mentioned, this study was limited in its abilities to measure the breadth and depth of subjective experiences pertaining to political polarization, as well as the complex motivations guiding intergroup perceptions and behaviors. Here, we measured subjective experiences via validated self-report measures of intergroup emotions and attitudes; however, such methods impose theoretical assumptions about the nature of subjective experiences. This limitation can potentially be addressed through transdisciplinary methods, described as a synthesis of qualitative and empirical approaches. A central principle of transdisciplinary mixed methods is that complicated phenomena cannot be fully understood through reductionist approaches but rather necessitate inquiry of the meaning individuals attribute to such phenomena ^95^. Reactions to political events are in part shaped by partisan identities, but they are also shaped by racial/ethnic identities ^96–98^, beliefs about equity and freedom ^99,100^, and moral narratives communicated via mass media ^101^. By interviewing participants about their identities, belief systems, and their understandings of political media, we may begin to grasp how constellations of meaning arise from biopsychosocial dimensions of emotion.

Limitations related to the intervention programs, featuring mindfulness-based and cognitive reappraisal-based training, should also be considered. Researchers theorize that the mechanisms of mindfulness and cognitive reappraisal are not mutually exclusive ^102,103^, and studies directly comparing mindfulness and cognitive reappraisal have reported their equal impact on managing negative emotions ^104,105^. These similarities pose a problem for the present study, which did not include a passive control group (i.e., waitlist) due to funding limitations. Thus, the mechanisms and salutary effects of mindfulness and cognitive reappraisal may be difficult to disentangle absent comparison against a passive control.

It warrants noting that we did not assess ingroup or outgroup attitudes at baseline in order to conceal the genuine objective of the study: to test the effects of training political intergroup emotions and attitudes. This decision was intended to reduce bias associated with demand characteristics ^40^; however, it bears the limitation of obscuring sources of within-person variability. While it is possible that both training programs reduced outgroup prejudices, it is equally possible that neither training program conferred prosocial benefits, as suggested by the relatively high levels of negative outgroup attitudes. Scholars continue to debate the extent to which mindfulness training can promote prosocial behavior ^40,41,106^, and it possible that such effects are only detectable at higher doses of mindfulness training, as illustrated by studies reporting reduced intergroup prejudice following 8 weeks of MBSR compared against a passive control. ^34,35^

The present study was exploratory in nature with little precedent for hypothesis testing or sample size estimation. Thus, conclusions drawn from this study are limited by a small sample size, which was based on power estimations to detect between-group effects in neural synchrony. Finally, the study design may also present limitations to generalizability, given that the study population included only Democratic-voting, liberal-leaning participants in order to limit sources of variability attributed to party-based ideological differences.

### Conclusion

This project was motivated to clarify the biopsychosocial mechanisms contributing to political polarization while evaluating the potential for mindfulness training to target partisan intergroup emotions and biases. Animus between U.S. Republican and Democratic partisans continue to escalate during a historical moment in which bipartisan action is increasingly critical for overcoming social, ecological, and financial crises (e.g., novel pandemics, climate change, global conflict, etc.). Political polarization however, undermines political power by decreasing trust in and compliance with public authorities ^107^, while increasing preference for antidemocratic policies ^108^ and avoidance of intergroup cooperation ^109,110^. This problem is complex, and cannot be fully understood or rectified using the research reported here. However, it is our hope that this research will open the door for continued interdisciplinary investigations to creatively examine and resolve complex social issues.

## Methods

### Participants

The study design and hypotheses were pre-registered with clinical trials identifier NCT04190030 (09/12/2019) and OSF registries (https://osf.io/htdc7; 07/07/2021). All study procedures were approved by the Virginia Commonwealth University Institutional Review Board and were performed in accordance with the institute’s guidelines and regulations. Data collection took place between July 2021 and June 2022. Given the novelty of this line of research, power for sample size determination was based on analyses of the proposed neural outcomes. Recent fNIRS research suggests that sample sizes of 60 – 75 are powered to detect two-group differences in PFC neural synchrony ^19,20^.

Participants were 72 healthy community adults recruited from the Richmond, Virginia area (see Table 4. for baseline characteristics). Prospective participants were screened for inclusion via an internet-administered survey. Inclusion criteria included proficiency in the English language, Democrat candidate-voting status, smartphone ownership (iOS or Android OS), absence of a new (non-acute) diagnosis of a medical or psychiatric condition within the last 3 months, and limited prior exposure to cognitive- or mindfulness-based training (practice < 2 times per week within the past 3 months). Prospective participants were excluded if they reported substance abuse/dependence or baseline stress levels <5 on the 4-item Perceived Stress Scale (PSS) ^111^. All participants provided written informed consent prior to enrollment in the study.

**Figure 5.**
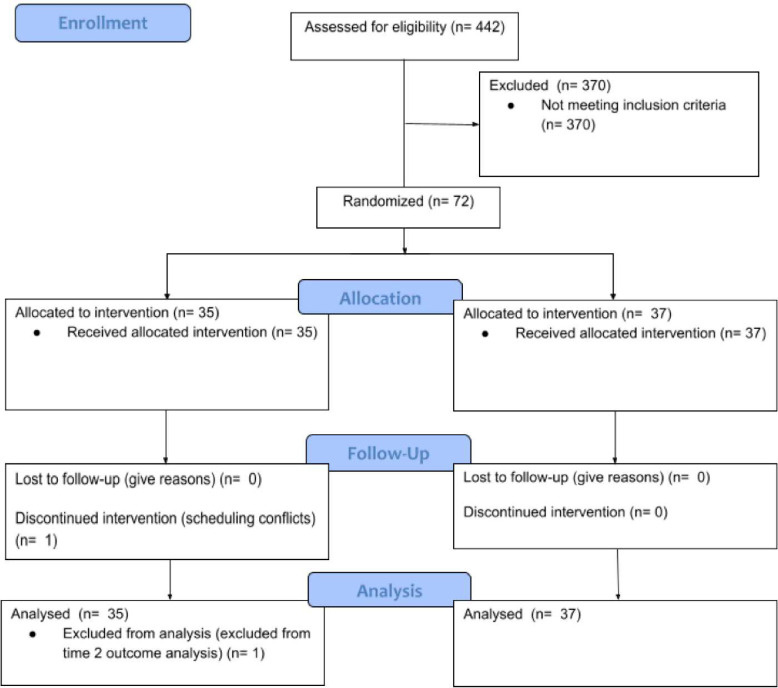
CONSORT flowchart.

Prior to data collection, condition randomization was conducted using block randomization (https://www.randomizer.org/) by a research team member who did not interact with any participant (KWB). Program allocations were written and stored in separate sealed envelopes labeled with a study ID number only. Program assignment was revealed to the participant in the first lab session following pre-training data acquisition, during which an undergraduate research assistant (RA) or graduate research assistant (GRA) opened the appropriate envelope. Program assignment was then recorded in an encoded dataform and the envelope was destroyed. See Figure 5. for a CONSORT flowchart.

To introduce participants to their training program and to equalize training expectancies, each participant viewed the same 5-minute introductory video explaining how to prepare for and what to expect in their training program. Immediately after viewing the video, each participant completed a brief self-report survey of training expectancies, the Credibility Expectancy Questionnaire (CEQ; Devilly & Borkovec, 2000). Preliminary analysis determined that MT and CT groups did not differ significantly in credibility/expectancy, *t*(70) = .542, *p* = .589.

### Procedure

Following successful enrollment, participants completed a baseline lab visit, including study orientation, provision of informed consent, and completion of self-report questionnaires assessing individual differences in emotion regulation. Hemodynamic responses were then recorded via fNIRS while participants underwent a naturalistic viewing task. Upon completing all baseline measures, participants were randomized to one of two structurally equivalent 14-day digital interventions (MT or CT). Following training completion (< 3 days after completing final lesson), participants attended a second lab session, during which they again underwent continuous fNIRS recording to assess cortical hemodynamic responses during a naturalistic viewing paradigm. Finally, participants completed a survey packet through which explicit attitudes towards political outgroup members were assessed.

### Naturalistic Viewing Paradigm

The present study adapted an ecologically valid viewing paradigm ^7^ in which participants viewed a series of inflammatory political partisan videos selected from publically available video streaming sources. All videos were prepared and validated for emotionality using the following procedure. Video editing software was used to edit video clips to a duration of 1–3 minutes and conceal logos shown on screen (given the potential for network or product logos to bias participant responses). A total of 10 experimental and 10 control video stimuli were prepared and examined for validity. Stimulus validation was assessed using a sample of 203 Democratic-voting U.S. citizens recruited through Prolific (prolific.co). Participants passively viewed and rated all video clips for emotionality (i.e., arousal, pleasure) using a sliding scale (0–100). Exploratory factor analyses (EFA) were conducted to identify videos fitting a two-factor structure (i.e., experimental and control). Prior to analyses, variables were checked for univariate and multivariate normality and outliers of +/− 3 SD were winsorized. Two EFAs were performed using a Promax rotation including 12 items to assess the structure of emotional arousal and 12 items to assess the structure of emotional (dis)pleasure. Inspection of scree plots and factor loadings suggested a two factor structure with 8 items loading meaningfully onto each factor (eigenvalues exceeding .50) (DeVellis & Thorpe, 2021). Thus, we identified 8 experimental and 8 control videos; half of which were presented at the pre-training lab and the other half were presented at follow-up.

The naturalistic viewing task consisted of four emotionally neutral and four emotionally negative video clips (approximately 1–3 minutes in length), which were block-order randomized (at the participant-level) and presented to participants sequentially. Audio was delivered via headphones. Immediately following each video, participants rated emotion reactivity across five emotions—joy, anger, fear, disgust, sadness—via a digital affective slider (scaled 0–100) ^114^. To test the specificity of the stimuli on emotional reactions and to reduce pre-post-training carryover effects, the political video stimuli were embedded in a brief series of neutral video stimuli. Video order randomization, stimuli delivery and behavioral data acquisition were completed using PsychoPy^®^ software ^115^.

### Functional Near-Infrared Spectroscopy (fNIRS)

**Figure 6.**
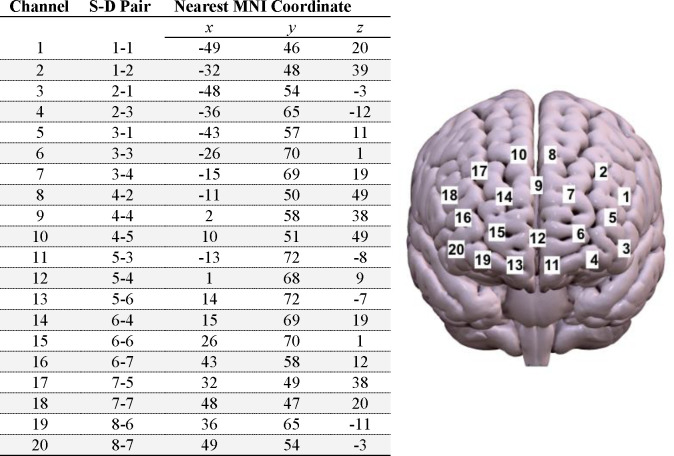
Estimated MNI coordinates and 3D cortical locations of each fNIRS channel.

Neural responses to video stimuli were assessed using fNIRS (NIRSport imaging unit from NIRx; nirx.net/nirsport), a neuroimaging modality suited to detect hemodynamic response as a spatially-sensitive indicator of brain function. Spatial positioning of light sources and detectors was standardized using the 10–10 UI external positioning system and light intensity data was collected at wavelengths of 760 and 850 nm and a sampling rate of 7.8 Hz. An elastic cap was used to affix eight light sources and eight detectors positioned according to a 20-channel prefrontal cortical montage, optimally suited for detecting activation from dorsolateral and medial prefrontal cortical structures. Positioning of nodes and approximate anatomical location of each channel in 3D cortical space are displayed in Figure 6. NirsLAB software was used to test optode saturation levels and ensure signal quality prior to data acquisition.

### Interventions

The Mindfulness Training and Active Coping training programs were developed and validated as part of a three-pronged randomized controlled trial that aimed to isolate monitoring and acceptance components of mindfulness while controlling for nonspecific training features ^64,116^. Both interventions were structurally equivalent and delivered by the same instructor. Each program included daily audio lessons of 15–20 minutes in length and daily brief, experiential homework assignments (3–10 minutes per day). Each audio lesson trained specific techniques through didactic explanation, guided practice, and self-guided practice. Research assistants contacted participants by phone on days 3 and 9 of the intervention program to address difficulties or training-specific questions and encourage participant adherence. Research assistants also monitored daily progress through the program to ensure lesson compliance, and participants were encouraged to text or call the study hotline to ask questions or resolve technical issues. If participants failed to complete a lesson, they were instructed to complete the previous day’s lesson before continuing with the scheduled lesson. Participants who missed two consecutive lessons were instructed to follow a two-lesson schedule for two days. Participants who missed three consecutive lessons were contacted to determine possible discontinuation from the study. Using this procedure, 71 of 72 participants completed the full 14-day lesson schedule.

#### Mindfulness Training (MT)

Mindfulness participants first learned foundational concentration skills that enabled them to (1) monitor their present-moment body experience (in the lessons, this skill was referred to as ‘sensory clarity’) while (2) welcoming and accepting each experience (referred to as ‘equanimity’). Monitoring (‘sensory clarity’) was explained in terms of two dimensions: resolution (discriminating types of experiences; e.g., pleasant, unpleasant, neutral; physical vs. emotional) and sensitivity (i.e., detecting subtle sensations). Acceptance (‘equanimity’) was trained through three tangible strategies that embodied the attitude of acceptance: participants were encouraged to (a) maintain a state of global body relaxation, (b) mentally welcome all physical and emotional body experiences, and (c) use a gentle, matter-of-fact tone of voice (an ‘equanimity tone’) while labeling these experiences.

#### Active Coping Training (CT)

The active coping program was developed to parallel the structure of Mindfulness training without encouraging focus on or acceptance of present experience. Instead, participants were instructed to reframe or reappraise past and anticipated events (with past and future emphasis contrasting present-focused monitoring, and change strategies contrasting acceptance strategies), and analyze and solve personal problems (again encouraging active change rather than acceptance of momentary experiences). The active coping program was designed to be useful for managing stress (reinforcing common reappraisal and problem solving strategies) without promoting mindful emotion regulation strategies.

### Behavioral Outcomes

#### Discrete emotions.

Immediately after each video, the Discrete Emotions Questionnaire ^117^, delivered via the validated Affective Slider digital scale ^114^, was used to assess anger, disgust, fear, sadness, and joy. The sliding scale was presented on screen with the anchors, 0 (no emotion) to 100 (an extreme amount of emotion), and tick marks placed at 10 point increments. Participants were allotted 5 seconds to rate each emotion before proceeding to the next scale. To ensure understanding of and compliance with the procedure, participants completed a practice round in which they viewed and rated emotional reactions to the classic Charlie Chaplin ‘Roller Skating’ scene from the film, *Modern Times*.

#### Intergroup attitudes.

Attitudes towards political ingroup (i.e., Democrat) and outgroup (i.e., Republican) members were measured using an affective prejudice measure and a social distancing scale. Affective prejudice towards Democrat and Republican group members was assessed using a validated sliding scale ^118^, in which participants rated feelings of warmth towards target groups on a scale of 0 (cold/unfavorable) to 100 (very warm/favorable). Target groups included Democrats, Republicans, and distractor groups (Americans, undocumented migrants, and Europeans). The social distancing scale, adapted from Moore-Berg et al. (2020) examined desire to remain separate from political outgroup members. Participants answered three items to indicate how comfortable they would feel if a political outgroup member was their doctor, their child’s teacher, or their child’s best friend. The sliding scale ranged from 0 (not at all comfortable) to 100 (very comfortable). Thus, higher scores reflected lower desire for social separation.

### Data Analyses

#### fNIRS preprocessing

Neural time courses for each video were trimmed and concatenated by video type, resulting in 2 neural time courses for concatenated political and neutral video clips. Raw fNIRS data were preprocessed using a Matlab wrapper function (S. Burns & Lieberman, 2019; MIT License) with Homer2 analysis package dependencies ^119^. The preprocessing pipeline first trimmed the time course to remove additional scan time before or after the presentation of stimuli. Then, channels with excessive noise were identified and channels were labeled “unusable” if detector saturation occurred for more than 2 seconds or if the signal’s power spectrum resembled white noise (i.e., the quartile coefficient of dispersion < .1). NIRS data were then filtered using a bandpass filter of .005-.5 Hz and were corrected for motion artifacts via a PCA algorithm. The resulting signals were converted to hemoglobin concentrations relative to baseline using the Modified Beer Lambert Law and z-scored. Finally, a Pearson’s correlation was used to examine remaining measurement errors among signals of each channel. Neuroimaging analyses were conducted on standardized total oxygenated-deoxygenated hemoglobin (HbO - Hb) concentrations. After exclusion of data with excessive channel noise, data from 64 participants (MT n = 31; CT n = 33) were used for neural synchrony-based analyses.

A probabilistic registration method ^120^ was used to estimate approximate MNI coordinates for each channel position. This method has previously been used to localize fNIRS data to common 3D brain space, thus enabling cross-modal comparison with data obtained through fMRI ^121^. Data was converted to *img files using xjView and overlaid on a 3D cortical surface via Surf Ice software.

#### Neural Synchrony Approach

An intersubject correlation (ISC) approach was used to determine if training groups exhibited significantly different patterns of neural synchrony while viewing highly emotional politically partisan videos. ISC is a data-driven technique developed to identify neural regions in which activity systematically fluctuates for participants exposed to the same time-locked stimulus ^23^. Within a single subject, activity in a neural region, $X_{A}(t)$, may be considered a combination of activation commonly shared across participants, $\alpha_{A}C(t)$, idiosyncratic activity, $\beta_{A}id_{A}(t)$, and noise driven by indeterminate sources, $\varepsilon_{A}(t)$. This relationship is represented with the formula:

$$X_{A}(t)=\alpha_{A}C(t)+\beta_{A}id_{A}(t)+\varepsilon_{A}(t)$$

Shared activity, or s*ynchrony*, can be estimated by averaging $X_{A}(t)$ between many pairs of subjects, producing a subject-by-subject correlation matrix. Regions with significant time-locked synchrony can be inferred as relevant for shared information processing, ranging from basic sensory perceptions to the interpretation of complex social emotional stimuli.

While such a *pairwise approach* is recommended as the first-level analyses prior to one-sample group-level analyses, a variation of this approach—referred to as a *leave-one-out approach* is ideal for two-sample tests ^122^. In contrast to a pairwise approach, a leave-one-out approach estimates individual-level ISC values $\left(X_{A}\right)$ using the average time course of every subject *with the exception of the subject’s own time course data*. Accordingly, a given group’s ISC value may be described as:

$$ISC_{Group}\sim r{\left(X_{A},{\underline{X}}_{Group\neq A}\right)}^{2}$$

Given that the aim of this study is to identify group-level differences (i.e., mindfulness *versus* active coping trainees) in neural synchrony, ISCs for each channel were calculated using a leave-one-out approach ^123^.

Group-level inferential testing is complicated due to intercorrelations of ISC coefficients, which violate assumptions of statistical independence ^65^. To address this concern, Chen et al. (2016) conducted simulation analyses to test the statistical validity of a series of non-parametric approaches with respect to controllability of false positive rates (FPR) and power. Accordingly, Chen et al. (2016) recommended that between-group comparisons be tested *indirectly* by comparing the difference between within-group ISC and between-group ISC matrices:

$$H_{0}\;:ISC_{within}=ISC_{between}$$

This may be accomplished through subject-wise permutation (SWP), which compares centrality of observed data to that of a null distribution, generated by randomly reassigning group membership over a number of iterations (typically 5000). In accord with recommended procedures for FPR controllability, subject-level hypothesis testing was conducted using SWP ^65^ in order to identify channels with significant within-group synchrony (one-sample analyses) and significant within- *versus* between-group synchrony (two-sample analyses).

ISC may be leveraged to capture brain activity driven by a time-locked stimulus, even when such activity reflects nuanced interpretations of complex social-emotional information ^18^. However, the nature of such interpretations remains ambiguous without statistical approaches suited to detect brain-behavior relations. This limitation may be accounted for by adapting the logic of ISC to an individual differences framework, an approach referred to as Intersubject Representational Similarity Analysis (IS-RSA) ^75^. Given that behavior-dependent signal may be derived from idiosyncratic activity, $\beta_{A}id_{A}(t)$, IS-RSA is positioned to triangulate sources of idiosyncratic neural signal. More specifically, IS-RSA compares (dis)similarity structures of brain and behavior data, operationalized as the Euclidean distance between each pair of subjects’ time courses or behavioral scores. Where $c_{1}(t)$ is the stimulus-evoked response for subject 1 and $c_{2}(t)$ is the stimulus-evoked response for subject 2, a pairwise distance may be expressed as the following:

$$D=\sqrt{\Sigma_{t}{\left(c_{1}(t)-c_{2}(t)\right)}^{2}}$$

Iterated over all pairs of subjects, this calculation produces a Representational Dissimilarity Matrix (RDM) of intersubject Euclidean distances for the neural time course of each region and each behavioral measure of interest. It warrants noting that Euclidean distance metrics assume a particular brain-behavior similarity structure in which subjects rank-ordered by behavioral scores are most similar to their immediate neighbors ^75^. This structure is referred to as a Nearest Neighbor (NN) model and may be contrasted with an Anna Karenina (AnnaK) model which assumes that brain-behavior similarities increase monotonically. Thus, while a NN model uses a Euclidean distance metric, an AnnaK model uses a distance metric based on absolute position (e.g., the mean of two subjects’ rank divided by the number of subjects). Determining which similarity structure (and by extension, distance metric) is most appropriate is accomplished by conducting IS-RSA with both NN and AnnaK models and inspecting models for differences in representational similarity, either statistically or visually. For example, the distribution of brain-behavioral similarities by region may be visually compared by histogram and scatter plot, or the mean of both distributions may be compared via paired-sample t-test. Finally, hypothesis testing is performed by correlating the upper triangles of brain and behavioral similarity matrices and conducting subject wise permutation (SWP), as recommended for FPR controllability ^75^.

In the present study, inter-subject representational similarity analyses (IS-RSAs) were used to determine if neural similarities were indicative of shared social-emotional experiences or perceptions of political outgroup members. First subject-by-subject inter-subject similarity matrices were calculated from fNIRS time courses and discrete emotion ratings (i.e., joy, anger, fear, sadness, and disgust). Similarities in the structures (of variations) of behavioral pairwise correlations and neural ISC were examined using a Mantel test. Nearest neighbor (NN) and Anna Karnina (AnnaK) models were then compared for best fit using a paired-samples t-test and scatterplot examination. Finally, we tested for significant neural representation of behavioral scores using non-parametric hypothesis testing with 5000 permutations (*p* = .05, *k* = 5).

#### Evaluating Training-Based Effects

**Figure 7.**
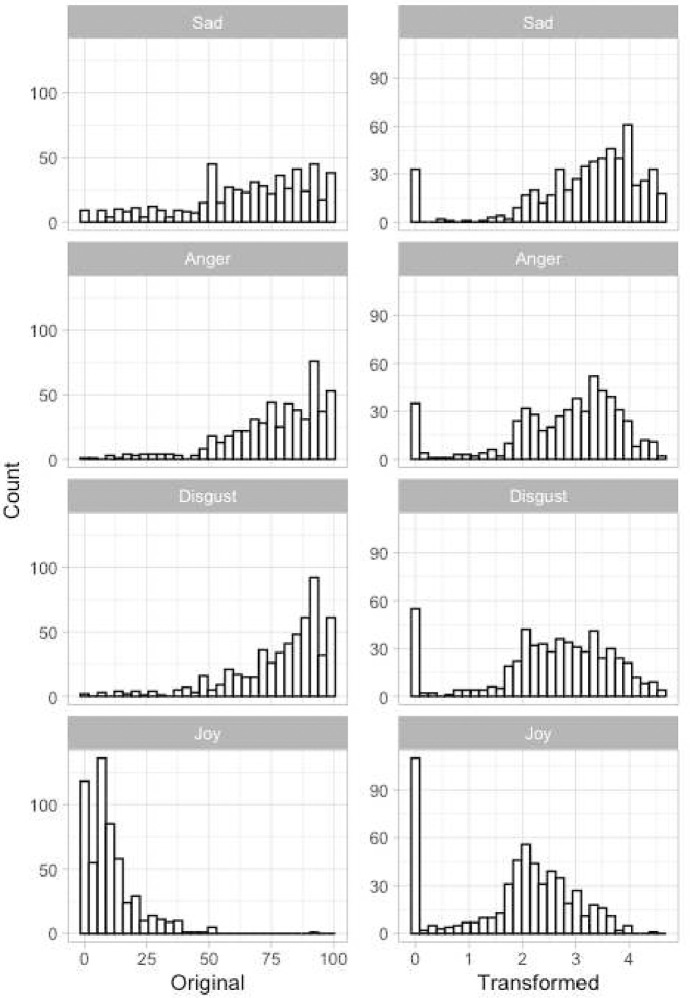
Histograms of original and transformed outcomes

To test hypotheses for the second aim of the study—specifically, whether mindfulness training (MT) would diminish negative emotional reactions and amplify positive emotional reactions to the videos—we employed a series of mixed models. These models incorporated time (0 = pre-training, 1 = post-training), group (0 = CT, 1 = MT), and their interaction for each outcome. Addressing notable skewness in sadness, anger, disgust, and joy, as well as floor and ceiling effects, we implemented a sequence of data transformations. For negative outcomes (sadness, anger, disgust), transformations were applied, such that lower values indicated more intense emotions, while higher values indicated less intense emotions. Subsequently, all outcomes were recalibrated to have 1 as the lowest value and were then log-transformed. The distributions of initial and transformed outcomes are illustrated in Fig. 7. We did not apply any transformations on fear as its distribution did not have the above-mentioned issues.

This series of transformations served to separate extreme responses (the highest possible values of negative emotions and zero joy) from less extreme responses. Zero-inflated Gaussian mixed models ^124^ were employed to accommodate such outcomes, segregating zero and Gaussian-distributed positive values into two submodels. The first submodel constituted a logistic mixed model fitted using Penalized Quasi-Likelihood to estimate the probability of the outcome being zero (extreme response) or a positive value (non-extreme response). The second submodel was a linear mixed model fitting the non-extreme responses using Maximum Likelihood. To model mindfulness training effects on fear, we estimated a single Restricted Maximum Likelihood mixed model that did not contain a zero-inflated part. All these models accounted for within-subject correlation, a characteristic of longitudinal data. We used R version X, *NBZIMM* R package version X (Zhang & Yi, 2020) to estimate two-part zero-inflated mixed models, *nlme* R package version X ^125^ to estimate a mixed model for fear, and *performance* R package version X ^126^ to estimate intraclass correlation coefficient (ICC), as well as marginal and conditional R^2 127^.

## Figures and Tables

**Table 1.** Mindfulness training effects on sadness, anger, disgust, and joy

	Sadness			Anger			Disgust			Joy		

*Predictors*	*B*	*95% CI*	*p*	*B*	*95% CI*	*p*	*B*	*95% CI*	*p*	*B*	*95% CI*	*p*

**Gaussian Submodel**												
(Intercept)	3.36	3.17 – 3.57	**<.001**	2.78	2.57 – 3.00	**<.001**	2.56	2.35 – 2.79	**<.001**	2.28	2.08 – 2.46	**<.001**
Time	0.08	−0.06 – 0.22	.279	0.22	0.07 – 0.38	**.004**	0.36	0.22 – 0.51	**<.001**	−0.20	−0.35 – −0.05	**.010**
MT group	−0.13	−0.42 – 0.16	.388	−0.05	−0.36 – 0.26	.742	−0.03	−0.35 – 0.28	.835	−0.11	−0.38 – 0.16	.426
Time × MT group	−0.05	−0.25 – 0.15	.628	0.21	−0.01 – 0.42	.065	0.05	−0.16 – 0.26	.638	0.25	0.04 – 0.46	**.018**
**Random Effects**												
σ^2^		0.35			0.38			0.35			0.32	
τ_00_		0.28			0.32			0.35			0.23	
ICC		0.444						.498			.422	
Marginal R^2^ / Conditional R^2^		.010 / .450			.040 / .480			.051 / .523			.010 / .428	
**Zero-Inflated Submodel**		Sadness				Anger			Disgust			Joy

*Predictors*	*OR*	*95% CI*	*p*	*OR*	*95% CI*	*p*	*OR*	*95% CI*	*p*	*OR*	*95% CI*	*p*

(Intercept)	0.01	0.00 – 0.02	**<0.001**	0.01	0.01 – 0.04	**<0.001**	0.02	0.01 – 0.05	**<0.001**	0.07	0.03 – 0.15	**<0.001**
Time	1.98	1.00 – 3.93	0.050	1.72	1.00 – 2.96	**0.049**	1.35	0.80 – 2.28	0.261	3.82	2.31 – 6.33	**<0.001**
MT group	2.71	0.63 – 11.59	0.178	1.50	0.40 – 5.66	0.543	2.99	0.77 – 11.58	0.113	1.34	0.43 – 4.14	0.606
Time × MT group	0.58	0.24 – 1.42	0.238	0.11	0.04 – 0.29	**<0.001**	0.08	0.03 – 0.18	**<0.001**	0.16	0.08 – 0.35	**<0.001**
**Random Effects**												
σ^2^		3.29			3.29			3.29			3.29	
τ_00_		4.69			4.27			5.20			3.70	
ICC		.588			.565			.613			.529	
Marginal R^2^ / Conditional R^2^		.024 / .598			.062 / .591			.071 / .640			.048 / .552	

N_ID_		72			72			72			72	
Observations		562			542			565			568	

*Note.* B = unstandardized regression coefficient; OR = odds ratio; CI = confidence interval; MT = mindfulness training; σ^2^ = level-1 residual variance; τ_00_ = variance in individual intercepts; ICC = intraclass correlation coefficient; N_ID_ = number of participants; marginal R^2^ = proportion of variance in the outcome explained by fixed effects only; conditional R^2^ = proportion of variance explained by fixed and random effects together.

**Table 2.** Mindfulness training effects on fear

Predictors	B	95% CI	p

(Intercept)	51.63	43.85 – 59.41	**<.001**
Time	−0.04	−4.27 – 4.18	.984
MT group	−6.59	−17.90 – 4.72	.249
Time × MT group	5.76	−0.32 – 11.83	.063
**Random Effects**			
σ^2^		333.88	
τ_00_		492.76	
N_ID_		72	
Observations		564	
ICC		.596	
Marginal R^2^ / Conditional R^2^		.009 / .600	

*Note.* B = unstandardized regression coefficient; CI = confidence interval; MT = mindfulness training; σ^2^ = level-1 residual variance; σ_00_ = variance in individual intercepts; ICC = intraclas correlation coefficient; N_ID_ = number of participants; marginal R^2^ = proportion of variance in the outcome explained by fixed effects only; conditional R^2^ = proportion of variance explaine by fixed and random effects together.

**Table 3.** Intersubject representational similarity analysis (IS-RSA) by Training Group

	Cognitive Reappraisal Training	Mindfulness Training
**Anger**	Channel 19 (*r* = .157, *p* = .047)^a^	Channel 5 (*r* = .100, *p* = .037)^b^ Channel 6 (*r* = .110, *p* = .031)^b^
**Disgust**	Channel 12 (*r* = .152, *p* = .033)^a^ Channel 13 (*r* = .197, *p* = .021)^a^ Channel 19 (*r* = .223, *p* = .0034)^a^	Channel 12 (*r* = .115, *p* = .021)^b^
**Sadness**	Channel 12 (*r* = .091, *p* = .049)^b^	Channel 18 (*r* = −.150, *p* = .049)^a^
**Fear**	—	—
**Joy**	Channel 4 (*r* = .129, *p* = .023)^b^ Channel 6 (*r* = .116, *p* = .0124)^b^	Channel 2 (*r* = −.145, *p* = .015)^a^ Channel 10 (*r* = −.132, *p* = .025)^a^

a Fitted to Anna Karenina (AnnaK) similarity structure

b Fitted to Nearest Neighbor (NN) similarity structure

**Table 4.** Participant Demographics

	Mindfulness (n = 35)	Active Coping (n = 37)	

	*M (SD)*	*M (SD)*	*P* ^1^
	
Age	28.29 (8.67)	27.30 (8.69)	.63
	*n (%)*	*n (%)*	*P* ^2^
	
**Gender**			
Cis-woman	31 (88.57)	26 (70.27)	.08
Cis-man	3 (8.57)	8 (21.62)	
Non-binary	1 (2.86)	3 (8.11)	
**Race/ethnicity**			
White	21 (60.00)	22 (59.46)	.56
Black/African American	5 (14.29)	5 (13.51)	
Hispanic or Latino	4 (11.43)	3 (8.11)	
East Asian	1 (2.86)	2 (5.41)	
South Asian	4 (11.43)	6 (16.22)	
Southeast Asian	1 (2.86)	2 (5.41)	
**Marital Status**			
Married	9 (25.71)	8 (21.62)	.91
Divorced	2 (5.71)	2 (5.41)	
Never Married	24 (68.57)	27 (72.97)	
**Annual Household Income**			
Less than $25,000	11 (31.43)	9 (24.32)	.52
$25,000 – $39,000	2 (5.71)	7 (18.92)	
$40,000 – $54,000	4 (11.43)	2 (5.41)	
$55,000 – $69,000	6 (17.14)	2 (5.41)	
$70,000 – $84,000	3 (8.57)	2 (5.41)	
$85,000 – $99,000	1 (2.86)	5 (13.51)	
$100,000 – $114,000	1 (2.86)	1 (2.70)	
$115,000 – $129,000	1 (2.86)	2 (5.41)	
$130,000 – $144,000	2 (5.71)	2 (5.41)	
$145,000 – $159,000	1 (2.86)	1 (2.70)	
$160,000 or more	3 (8.57)	4 (10.81)	
**Education**			
Graduated high school	1 (2.86)	2 (5.41)	.58
Some college/no degree	6 (17.14)	10 (27.03)	
Associate’s degree	4 (11.43)	1 (2.7)	
Bachelor’s Degree	14 (40.00)	15 (40.54)	
Post-graduate degree	10 (28.57)	9 (24.32)	

1 Significance value of two-sample t-test.

2 Significance value of Fisher’s Exact Test.

## Data Availability

The study design, procedures, and hypotheses were pre-registered with OSF registries (https://osf.io/htdc7) and are publicly available. We examined prepregistered aims 1–3, with specific aims 1 and 3 receiving support. Specific aim 2 proposed to examine if—relative to Cognitive Reappraisal Training (CT)—Mindfulness Training (MT) resulted in less affective prejudice and less social distancing. Because we decided to assess this outcome at post-training only, multilevel modeling (the proposed analysis approach) was no longer appropriate for analysis and we instead opted to use a Mann Whitney U test. Given that no between-group effects were observed, the influence of baseline traits was not reported here. Analysis of fNIRS data was conducted using scripts adapted from publicly available sources (https://naturalistic-data.org/content/intro.html).^119^ Scripts for behavioral data analysis may be made available upon request. Subject data will not be made publicly available but de-identified data may be shared with researchers upon request.
